# Cannabidiol (CBD) and cognitive function in older adults: a mini review

**DOI:** 10.3389/fpsyt.2025.1646151

**Published:** 2025-08-28

**Authors:** Alicja Anna Binkowska, Agnieszka Mateja, Natalia Jakubowska

**Affiliations:** ^1^ Institute of Psychology, Humanitas University, Sosnowiec, Poland; ^2^ Institute of Psychology, SWPS University, Warsaw, Poland

**Keywords:** cannabidiol, CBD, cognitive aging, neuroprotection, endocannabinoid system, older adults, cognition, aging

## Abstract

As the global population ages, the need for effective interventions to support cognitive health in older adults is growing. Cannabidiol (CBD), a non-intoxicating component of cannabis, has emerged as a potential neuroprotective agent due to its anti-inflammatory, antioxidant, and anxiolytic properties. While preclinical studies show promising effects on hippocampal neurogenesis and cognitive performance, human trials remain limited, particularly in older populations. Existing studies have focused primarily on young, healthy adults and acute administration, often using oral routes that yield low and variable bioavailability. Furthermore, the endocannabinoid system undergoes age-related changes, potentially altering CBD efficacy in older adults. This review synthesizes current evidence on CBD and cognitive function, emphasizing age as a moderating factor, exploring pharmacokinetic challenges, and identifying key research gaps. The review calls for well-controlled trials in older adults using standardized cognitive measures, neuroimaging, and biomarker assessments. Understanding the age-specific impact of CBD on cognition is essential for evaluating its therapeutic potential in an aging society.

## 1 Introduction

It is estimated that by 2050, approximately 16% of the global population will be over the age of 65 (1). The aging process is commonly accompanied by cognitive decline, including deteriorations in memory, attention, and executive function (2, 3). Although the extent and trajectory of cognitive aging vary significantly across individuals (4), even modest declines can adversely affect mental health, independence, and overall quality of life in older adults (5).

Given the limitations of current pharmacological options for preventing or mitigating age-related cognitive decline, there is growing interest in alternative compounds with neuroprotective potential. Cannabidiol (CBD), a non-intoxicating component of cannabis, has recently emerged as a promising candidate. Unlike delta-9-tetrahydrocannabinol (THC), CBD does not produce intoxicating effects and has been shown to have anti-inflammatory, anxiolytic, and antioxidant properties (6–8). CBD is considered to be well tolerated in humans and exhibits a favorable safety profile, even at relatively high doses (9, 10).

Cannabidiol exerts complex, multifaceted effects on the central nervous system, and its full mechanisms of action remain incompletely understood. While CBD has low affinity for classical cannabinoid receptors CB1 and CB2, it functions as a negative allosteric modulator of CB1 receptors, altering receptor conformation and attenuating CB1-mediated signaling without directly activating the receptor (7, 8). CBD also indirectly influences the endocannabinoid system by inhibiting FAAH (fatty acid amide hydrolase), thereby increasing endogenous levels of anandamide (7). Beyond the ECS, CBD interacts with a diverse range of non-cannabinoid targets. It partially activates GPR55, which modulates neurotransmission and may contribute to anticonvulsant effects (8). CBD also influences serotonin 5-HT1A receptors, though *in vitro* studies suggest this interaction may require higher concentrations than typically achieved *in vivo* (8). In addition, CBD acts on transient receptor potential (TRP) channels, such as TRPV1, and functions as a positive allosteric modulator of GABA-A receptors, mechanisms that may underlie its anxiolytic and neuroprotective effects (11). At the cellular level, CBD inhibits the equilibrative nucleoside transporter-1 (ENT-1), increasing extracellular adenosine concentrations and promoting anti-inflammatory signaling via A2A receptors (8). Furthermore, CBD modulates mitochondrial function and reduces oxidative stress, mechanisms implicated in preclinical models of neurodegeneration and age-related cognitive decline (12, 13).

These properties make it a potential candidate for mitigating cognitive decline associated with neurodegenerative conditions or aging-related brain changes (8, 12).

This review aims to provide an up-to-date overview of the evidence linking CBD with cognitive function, with a particular focus on older adults. It discusses recent findings, explores gaps in the literature, and identifies directions for future research.

## 2 Cannabidiol and cognitive function: current evidence

### 2.1 Preclinical evidence on neuroprotective CBD potential

A growing body of preclinical evidence suggests that CBD may mitigate age-related cognitive decline. In aging mice, Mirza et al. (13) reported that long-term oral administration of CBD (20 mg/kg daily for 7 months) reduced neuroinflammation and improved performance in hippocampal-dependent memory tasks such as novel object recognition and the Morris water maze. Similarly, Campos et al. (14) found that repeated injections of CBD (30 mg/kg, for 14 days) in chronically stressed mice increased the growth of new hippocampal neurons. This effect was mediated by CB1 and CB2 receptors and may have been driven by elevated endocannabinoid tone, due to inhibited anandamide breakdown. Additional evidence comes from a murine model of cerebral malaria, a severe complication of Plasmodium falciparum infection that can cause lasting neurological damage. Mice infected with *Plasmodium berghei* and treated with CBD (30 mg/kg/day) showed preserved memory and reduced anxiety-like behaviors, both during the peak of the disease and after recovery (15). CBD treatment increased brain-derived neurotrophic factor (BDNF) in the hippocampus and reduced proinflammatory cytokines, suggesting a dual anti-inflammatory and neurotrophic mechanism (15). CBD exerts anti-inflammatory, antioxidant, and neurogenic actions through multiple mechanisms. For example, Schouten et al. (11), in their review, report that in Alzheimer’s disease models, CBD’s neuroprotective effects may involve activation of peroxisome proliferator-activated receptor gamma (PPAR-γ) and inhibition of Nuclear Factor-Kappa B (NF-κB) - a key regulator of neuroinflammation although this mechanism has not yet been confirmed in non-Alzheimer’s aging models. This mechanism may protect against chronic inflammatory processes linked to cognitive decline. In addition, Luján and Valverde (16) reviewed preclinical studies demonstrating that CBD promotes adult hippocampal neurogenesis primarily by facilitating neuronal differentiation and increasing the survival of newborn neurons. These neurogenic effects are believed to underlie some of CBD’s behavioral benefits, such as its anxiolytic and antidepressant properties, and may contribute to cognitive resilience. The proposed mechanisms involve the upregulation of brain-derived neurotrophic factor (BDNF), modulation of PPAR-γ signaling, and interactions with the endocannabinoid system, though further clarification is needed.

In addition, preclinical studies suggest that CBD’s antioxidant effects may contribute to neuroprotection. Ni et al. (12) reported that CBD can reduce reactive oxygen species (ROS), help preserve mitochondrial structure and function, and mitigate oxidative stress–related cellular damage, including in neuronal models, although these effects can be bidirectional and may vary depending on dose, exposure time, and cell type. CBD has also been shown in some preclinical models to activate autophagy-related pathways, which may contribute to the clearance of damaged cellular components and support neuronal health during aging (12). Supporting this, Mirza et al. (13) observed that chronic CBD administration in aging mice reduced astrocyte-mediated inflammation in the hippocampus, which was accompanied by improvements in object recognition and aspects of spatial memory, potentially via adenosine-mediated anti-inflammatory signaling.

Collectively, these findings suggest that CBD may offer neuroprotective benefits through a combination of anti-inflammatory, antioxidant, autophagy-related and neurogenic pathways, although translational studies in humans remain limited and the observed effects are often dose- and model-dependent.

### 2.2 Effects on brain function in healthy adults

Evidence from neuroimaging studies indicates that CBD may affect cerebral blood flow and brain network connectivity. Bloomfield et al. (17) found that a single oral dose of 600 mg of CBD increased cerebral blood flow in the hippocampus in healthy young adults. The hippocampus is critical for memory, and previous research has linked greater hippocampal perfusion with better memory performance (18). However, Bloomfield et al. (17) did not observe corresponding behavioral effects on memory tasks, possibly due to ceiling effects in their cognitively intact sample (young healthy adults).

Similarly, Grimm et al. (19) reported that oral administration of 600 mg of CBD enhanced fronto-striatal connectivity in resting-state fMRI scans. This network plays a crucial role in executive function and decision-making. These findings collectively suggest that CBD may influence neural pathways underlying cognitive performance, although behavioral confirmation remains limited. While functional neuroimaging studies have begun to characterize changes in cerebral blood flow and connectivity, mechanistic insight at the molecular level in humans remains sparse. Future studies should include biomarkers of neuroplasticity, inflammation, or stress response to clarify how CBD exerts its effects on the aging brain.

### 2.3 Age as a potential moderator of CBD’s cognitive effects

Age appears to modulate the cognitive effects of CBD. In a recent study, Gebregzi et al. (20) administered 246 mg of CBD or a placebo to healthy adults. While no significant differences in learning and memory performance were observed between the groups overall, an age-related interaction emerged: older participants exhibited a trend toward improved cognitive outcomes with CBD. This suggests that aging may enhance sensitivity to CBD or involve distinct underlying mechanisms in this population. Despite these preliminary findings, research specifically targeting healthy older adults remains scarce. This represents a significant gap in the literature, particularly in light of the growing use of CBD among older individuals (21).

These observations align with evidence that the endocannabinoid system undergoes substantial age-related changes (22). Specifically, preclinical studies have shown that aging is associated with a progressive decline in CB1 receptor density and function in several brain regions critical for cognition, including the cortex, limbic system, hypothalamus, and hippocampus (23). In addition, the levels of endogenous cannabinoids such as 2-arachidonoylglycerol (2-AG) significantly decrease with age in the hippocampus, potentially impairing synaptic plasticity and memory processes (24). These alterations may affect how exogenous cannabinoids, including CBD, interact with the ECS in older adults. Although CBD does not directly activate CB1 receptors, it modulates ECS tone through mechanisms such as FAAH inhibition and anandamide elevation (7, 8). Age-related reductions in ECS signaling could therefore influence CBD’s efficacy, metabolism, and therapeutic profile in this population. Moreover, changes in receptor expression, enzyme activity, and endocannabinoid availability may alter pharmacokinetics and pharmacodynamics, highlighting the need for studies that explicitly examine age-dependent effects.

### 2.4 Route of administration and bioavailability

The route of administration significantly influences the pharmacokinetics and therapeutic effects of CBD. Many studies investigating CBD’s impact on cognitive function—particularly those conducted in clinical settings—have utilized oral administration, such as capsules or oil-based formulations. However, oral delivery is subject to first-pass hepatic metabolism, which substantially reduces systemic availability. Bioavailability following oral ingestion is typically low and variable, with estimates ranging from 6% to 19%, depending on factors such as formulation type, individual metabolic rate, diet, and age (25, 26).

Alternative routes, such as inhalation (vaporization), transmucosal or oromucosal delivery systems (e.g., sublingual oils, oromucosal sprays, dissolvable oral strips), and nasal administration, may offer more efficient and rapid absorption by partially or fully bypassing first-pass hepatic metabolism (25). These routes can lead to higher or more consistent plasma concentrations compared to oral ingestion, although data in older adults remain sparse.

Given that older adults often experience polypharmacy and altered drug metabolism, transmucosal and oromucosal delivery routes may offer practical advantages, including more predictable absorption, easier administration, and potentially fewer drug–drug interactions. This is particularly relevant because CBD, especially when administered orally, can inhibit cytochrome P450 enzymes (e.g., CYP3A4, CYP2C19), increasing the risk of drug–drug interactions with common medications such as anticoagulants, benzodiazepines, or antidepressants (27–29). Non-oral routes that bypass first-pass metabolism may reduce, though not eliminate, these risks. To date, no studies have directly compared multiple routes of administration in older adults, limiting our understanding of which method may maximize cognitive benefits while minimizing risks in this demographic. Future research should systematically examine oral, inhaled, and transmucosal/oromucosal routes to determine optimal strategies for cognitive support in aging populations.

## 3 Research gaps and emerging directions

Although CBD has gained attention for its potential cognitive benefits, particularly due to its neuroprotective and anti-inflammatory properties, significant research gaps persist. Notably, most existing studies have been conducted in young, healthy individuals, leaving a critical gap in understanding how CBD affects older adults—those most vulnerable to cognitive decline and potentially most likely to benefit from intervention—yet also physiologically distinct in ways that may alter drug metabolism, receptor sensitivity, and blood-brain barrier permeability (20, 22). In addition, aging is associated with declines in endocannabinoid tone, including reductions in CB1 receptor density and lower levels of key endocannabinoids such as 2-AG and anandamide, particularly in brain regions involved in memory and cognition (23, 24). These changes may influence how older adults respond to exogenous cannabinoids like CBD, further underscoring the need for age-specific research.

While neuroimaging studies have demonstrated alterations in brain function, such as increased hippocampal blood flow and enhanced fronto-striatal connectivity (17, 19), these changes do not consistently correspond with improvements in behavioral or cognitive outcomes. The absence of comprehensive cognitive testing and longitudinal follow-up further limits conclusions about sustained benefits.

Additionally, methodological inconsistencies across studies—including wide variations in dosing (ranging from 250 mg to 600 mg), routes of administration, and outcome measures—complicate comparisons and the formulation of best practices. Moreover, age-related physiological changes may alter pharmacokinetics, necessitating age-specific dosing guidelines to balance efficacy and safety - particularly given the potential for drug–drug interactions in polypharmacy-prone older adults. The risk of interactions is especially relevant for oral CBD products, as CBD can inhibit cytochrome P450 enzymes (CYP3A4, CYP2C19), affecting the metabolism of commonly prescribed medications (27–29).

Research has also largely focused on acute administration, while the effects of chronic or sustained CBD use—arguably more relevant for long-term cognitive support—remain largely uninvestigated. Furthermore, optimal dose-response relationships for cognitive benefits in older populations are unclear. Some findings suggest lower doses may be more effective for certain individuals, while others may require higher concentrations (8, 10).

Another important gap in the literature is the near-complete absence of sex-specific analyses. Both endocannabinoid system function and age-related cognitive decline exhibit notable sex differences, likely influenced by hormonal fluctuations and receptor distribution (30). Given these biological distinctions, older men and women may differ in their responsiveness to CBD, both in terms of efficacy and side-effect profiles. Addressing this issue in future research will be critical for developing personalized interventions.

In addition to pharmacological approaches, combining CBD with behavioral interventions such as cognitive training, physical exercise, or mindfulness-based programs may enhance outcomes through synergistic mechanisms. Preclinical studies suggest that CBD may support neuroplasticity and hippocampal neurogenesis (15, 16), which could amplify the effects of interventions designed to stimulate cognitive function. For example, cognitive training has been shown to improve executive functioning and memory in older adults by promoting functional brain reorganization (31), and these effects may be potentiated by neuroprotective agents like CBD. Yet, to date, no published studies have examined the combined effect of CBD and behavioral interventions on cognition in aging populations. Future research should explore whether multimodal approaches can produce additive or synergistic effects on cognitive outcomes, particularly in individuals at elevated risk for decline.

Addressing these gaps through rigorously designed, longitudinal trials in age-diverse and specifically older populations is essential for advancing the therapeutic potential of CBD in cognitive aging.

## 4 Discussion

The emerging literature suggests that CBD holds promise as a therapeutic agent for supporting cognitive health, particularly in the context of aging. Preclinical studies highlight its neuroprotective, anti-inflammatory, and neurogenic effects, with several investigations demonstrating improved hippocampal-dependent memory and reduced neuroinflammation in aged rodents following chronic CBD administration (13, 14). These findings provide a mechanistic rationale for exploring CBD as a potential intervention for age-related cognitive decline in humans.

Human studies, though limited, suggest that CBD can modulate brain function. Increased hippocampal blood flow and enhanced fronto-striatal connectivity have been observed following acute oral administration of CBD (17, 19), and such changes are theoretically consistent with improved cognitive processing. However, behavioral evidence remains inconsistent, with most studies conducted in young, cognitively intact participants who may be less sensitive to treatment effects due to ceiling performance. This limits generalizability to older adults, who are more likely to experience cognitive impairment and may have different neural and metabolic responses to CBD.

A particularly noteworthy issue is the underrepresentation of older adults in CBD research. Aging is associated with a host of neurobiological changes, including reduced endocannabinoid tone, altered receptor distribution, and increased vulnerability to oxidative stress and inflammation (22). Specifically, studies have shown age-related reductions in CB1 receptor density and decreased levels of endogenous cannabinoids such as 2-AG in the hippocampus—both of which may affect cognitive processes and cannabinoid responsiveness (23, 24). These factors may render older individuals more responsive to CBD, or may necessitate specific dosing strategies tailored to their unique physiology. Preliminary data (20) point to possible age-related interactions with CBD, yet dedicated studies in older populations remain sparse.

Another important factor concerns the route of administration. Oral delivery has dominated existing trials, yet it suffers from low and variable bioavailability due to first-pass metabolism.While vaporized CBD may achieve higher plasma concentrations and faster onset of action, this method is not always feasible or desirable for older adults. Transmucosal and oromucosal routes, such as sublingual oils, dissolvable strips, nasal sprays, and oromucosal sprays, offer alternative delivery options that may bypass or reduce first-pass metabolism while providing more consistent absorption. These formats may be better tolerated by older individuals and warrant investigation in future trials. Understanding how these routes affect both pharmacokinetics and cognitive outcomes in aging populations is essential for clinical translation.

Compounding these limitations is a lack of consistency in dosing regimens and outcome measures. Moreover, few studies assess chronic administration, which may be more relevant for mitigating progressive age-related cognitive changes. Acute effects may not capture the full therapeutic potential or safety profile of long-term CBD use, especially in older adults who are more likely to use such compounds on a sustained basis.

There is also a critical need to incorporate multi-modal assessment strategies in future research. Combining cognitive testing with neuroimaging, blood biomarkers (e.g., inflammatory cytokines, BDNF), and even genetic profiling (e.g., polymorphisms in CB1, CB2, or 5-HT1A receptors) could help identify responder profiles and uncover mechanisms of action. Such an approach would not only clarify efficacy but also inform personalized interventions.

In addition to potential direct effects on cognitive performance, CBD may confer cognitive benefits indirectly by improving conditions that negatively affect cognition, such as sleep disturbances and anxiety. These factors are particularly relevant in older adults, where poor sleep and chronic stress are both common and strongly linked to memory and executive function decline. CBD has shown anxiolytic and sleep-enhancing properties in previous research (10, 32–36), suggesting that its cognitive impact may be partially mediated through these pathways. Future trials should include assessments of sleep quality, stress, and mood to better capture the full spectrum of CBD’s influence on brain health in older adults.

In sum, while current findings offer a compelling rationale for continued investigation, the field remains in its early stages. Advancing our understanding of CBD’s role in cognitive aging will require rigorous, age-specific research designs that distinguish between acute and chronic use, reflect real-world dosing practices, and account for individual variability in pharmacokinetics and responsiveness. Only through such targeted approaches can we determine whether CBD is a viable and effective intervention for preserving cognitive health in older adults.
